# The Efficacy of Doxycycline Treatment on *Mansonella perstans* Infection: An Open-Label, Randomized Trial in Ghana

**DOI:** 10.4269/ajtmh.18-0491

**Published:** 2019-06-03

**Authors:** Linda Batsa Debrah, Richard O. Phillips, Kenneth Pfarr, Ute Klarmann-Schulz, Vera Serwaa Opoku, Norman Nausch, Wellington Owusu, Yusif Mubarik, Anna-Lena Sander, Christine Lämmer, Manuel Ritter, Laura E. Layland, Marc Jacobsen, Alexander Yaw Debrah, Achim Hoerauf

**Affiliations:** 1 Kumasi Centre for Collaborative Research in Tropical Medicine (KCCR), Kumasi, Ghana;; 2 Department of Clinical Microbiology, Kwame Nkrumah University of Science and Technology, Kumasi, Ghana;; 3 Department of Medicine, Kwame Nkrumah University of Science and Technology, Kumasi, Ghana;; 4 Institute for Medical Microbiology, Immunology and Parasitology, University Hospital Bonn, Bonn, Germany;; 5 German Center for Infection Research (DZIF), Partner Site Bonn-Cologne, Bonn, Germany;; 6 Department of General Paediatrics, Neonatology, and Paediatric Cardiology, University Children’s Hospital, Heinrich-Heine University, Düsseldorf, Germany;; 7 Faculty of Allied Health Sciences of Kwame Nkrumah University of Science and Technology, Kumasi, Ghana

## Abstract

Treating *Mansonella perstans* is challenged by the low efficacy of registered antihelminthics. *Wolbachia* endobacteria provide an alternative treatment target because depletion results in amicrofilaremia in filarial infections with *Wuchereria bancrofti* and *Onchocerca volvulus* infections. This open-label, randomized study sought to confirm that i) *Wolbachia* are present in *M. perstans* in Ghana and ii) doxycycline treatment will deplete *Wolbachia* and cause a slow, sustained decline in microfilariae (MF). Two hundred and two Ghanaians with *M. perstans* infection were randomized into early (immediate) and delayed (6 months deferred) treatment groups, given doxycycline 200 mg/day for 6 weeks, and monitored for MF and *Wolbachia* levels at baseline, 4, 12, and 24 months after the study onset (= time of randomization and start of treatment for the early group). Per protocol analysis revealed that the median MF/mL in the early group declined from 138 at baseline to 64 at month 4 and further to 0 at month 12. In the delayed group, MF load did not change from a baseline median of 97 to 102 at month 4 but declined to 42 at month 12, that is, 6 months after receiving treatment, trailing the early group as expected. By month 24, both treatment groups had reached a median MF level of 0. After treatment, *Wolbachia* were depleted from MF by ≥ 1-log drop compared with baseline levels. We conclude that *M. perstans* in Ghana harbor *Wolbachia* that are effectively depleted by doxycycline with subsequent reduction in MF loads, most likely because of interruption of fertility of adult worms.

## INTRODUCTION

*Mansonella perstans* infection, a vector-borne disease transmitted by female midges of the genus *Culicoides*, is an infection that, although not officially listed by the WHO, is nevertheless to be considered a neglected tropical disease. It affects more than 100 million people mainly in rural areas of Central Africa, the Caribbean, and South America.^1,2^ Recent reports suggest high prevalence in Ghana.^3,4^ In the middle belt of Ghana, the overall prevalence was 32%, but some communities had prevalences of up to 75%. Contrary to the assertion that maximal infection rates (up to 90%) occur in children aged 10 to 14 years,^5^ our studies in Ghana showed prevalence peaks after 20 years in the Sene West and Atebubu districts in the middle belt,^4^ suggesting that age prevalence differs with overall endemicity as is the case with other filarial infections.

After microfilariae (MF) are taken up from *M. perstans*–infected individuals by the midge, infective larvae develop and are then transmitted to human hosts. Adult filariae persist in serous cavities and retroperitoneal tissues for years. Microfilariae released by female worms circulate in the peripheral blood of infected individuals.^5–7^ Clinical manifestations of *M. perstans* infections include a wide range of symptoms such as arthralgias, serositis, angioedema, pruritus, fever, and headaches, which are often subclinical.^5^ The severity of these clinical manifestations is, in most cases, not very obvious because *M. perstans* is often coendemic with other filarial parasites in the hosts, making it difficult to assign clinical symptoms specifically to *M. perstans*.^3,8^

*Mansonella perstans* infection is not covered by large-scale programs for the control of filarial diseases such as onchocerciasis and lymphatic filariasis. *Mansonella perstans* worms often occur in the same geographical areas^9^ and could be a confounding agent in relation to diagnosis and compliance assessment. It has been shown that *M. perstans* infection attenuates immune responses associated with severe malaria and protects against anemia.^10^ By contrast, we have shown that *M. perstans*–microfilaremic individuals are characterized by increased TH2 and regulatory cell populations concomitant with reduced systemic cytokine/chemokine and increased filaria-specific IgG4 levels,^11^ which might lead to increased susceptibility and worsened disease course of HIV, tuberculosis (TB), and malaria,^12–14^ and the lower efficacy of bacillus Calmette–Guérin vaccination against TB.^15^ Infection with another filarial nematode, *Wuchereria bancrofti*, has recently been shown to increase the risk of acquiring concomitant HIV infection by a factor of 2–3, depending on the age of the patient.^16^ Filarial nematodes that polarize host immunity toward humoral and T helper type 2–mediated immunity and to immune-regulatory responses (involving Treg cell responses) are special candidates for impeding vaccine-induced protective immunity.^11,15,17,18^ Whatever the clinical consequences of *M. perstans* infection, the lack of an effective treatment may, ultimately, be a drawback to health professionals and patients, particularly in light of the United Nations recent adoption of the Sustainable Development Goals (e.g., SDG #3—ensure healthy lives and promote well-being for all at all ages). These issues highlight the importance that more attention is paid to this neglected infection.

Drugs that are usually used against other filarial parasites—diethylcarbamazine (DEC), ivermectin, and albendazole—have shown very limited efficacy against *M. perstans*.^5,19–21^ Therefore, the discovery that *M. perstans* from Mali^22,23^ and Gabon^24^ were positive for *Wolbachia* is a major breakthrough.^6^ However, the efficacy of treatment with doxycycline has only been addressed in a study in Mali^23^ and not yet repeated in other countries. Confirmation of the efficacy of doxycycline to treat *M. perstans* is critical because there have been controversial data on the distribution of *Wolbachia* endosymbionts in *M. perstans*.^23–25^

Therefore, the aim of this study was to determine whether *M. perstans* worms in Ghana harbor *Wolbachia* and to demonstrate the efficacy of doxycycline 200 mg/day for 6 weeks in depleting the *Wolbachia* with analysis of subsequent filaricidal effects, to evaluate the usability of doxycycline in *M. perstans* treatment.

## MATERIALS AND METHODS

### Study population.

The study was conducted in eight communities in the Asante Akim North District of Ghana, namely, Nhyieso, Serebouso, Afrisere, Anokye Beemu, Dukusen, Bebuso, Abutantri, and Ananekrom. The study site was selected based on previous reports on the presence of *M. perstans* in the blood of patients with Buruli ulcer,^3^ and the district has been screened in detail as published previously.^4^

This study was approved by the Committee on Human Research Publications and Ethics of the School of Medical Sciences of the Kwame Nkrumah University of Science and Technology (KNUST), Kumasi (CHRPE/AP/276/14). The trial was registered at ClinicalTrials.gov (NCT02281643; registered on October 26, 2014).

All participants either signed or thumb-printed written informed consent forms. For participants younger than 18 years, informed consent was obtained from parents or legal guardians in addition to the assent of the children. Participants were free to drop out at any time of the study.

Participants aged between 10 and 55 years with a minimum body weight of 40 kg and good medical condition without chronic medication were considered eligible. The exclusion criteria were abnormal hepatic enzymes (above AST [0–60 U/L], γ-GT [7–61 U/L], and creatinine [56–119 μmol/L]) assessed by a Selectra Pro S biochemistry analyzer (ELITechGroup, Puteaux, France), pregnancy, breastfeeding, intolerance to the study drugs, and alcohol or drug abuse. Pregnancy tests were administered before enrollment, before treatment, and every 2 weeks during treatment. Participants with *W. bancrofti* microfilaremia were excluded from the trial. The area is not endemic for onchocerciasis.

### Study design.

The study followed a randomized open-label trial design with two treatment arms. Randomization codes were computer-generated. Participants in the early treatment arm received immediate treatment after randomization, whereas those assigned to the delayed arm had treatment deferred for 6 months. The deferred treatment served as a control for the early treatment. The deferred/staggered treatment design was chosen to control for changes in MF unrelated to the treatment, which may occur during the first 12 months of observation. Even though it was an open study and as such participants and trial clinicians were not blinded, the outcome assessors (MF counting and *Wolbachia* determination) were blinded to the treatment allocation.

All participants were administered 200 mg (2 × 100 mg) doxycycline daily for 6 weeks under direct observation by the trial clinician. Doxycycline was purchased from Pill Point Pharmacy registered to distribute doxycycline (http://placesmap.net/GH/Pill-Point-Pharmacy-Atwima-Brofoyedu-38715/) in Ghana. All the clinical conditions that occurred during the treatment period were recorded by the trial clinician. Adverse events (AEs) were monitored in the early and delayed groups during doxycycline treatment.

### Microfilaria assessments.

The finger-prick test was used to quickly identify MF-positive patients in the communities by direct microscopy of unstained MF in thick blood smears.^26^ From those who were screened positive, up to 5 mL of venous blood was collected into an EDTA vacutainer (BD Biosciences, Heidelberg, Germany) for MF load assessment first in a Sedgewick counting chamber and then by the filter method.^27^ Counting in a Sedgewick chamber served to obtain a good estimate on the MF load (MF/mL) in blood to allow calculation of a dilution factor for the blood for countable MF densities in the filter method. Filters were used for direct counting and for polymerase chain reaction (PCR) confirmation of *M. perstans* and for *Wolbachia* quantification.

For the membrane filter test, 0.1–1 mL of venous blood (according to MF loads assessed by Sedgewick counting) was filtered using a Whatman nucleopore membrane filter (VWR, Langenfeld, Germany) with a pore size of 3 μm. Microfilariae that were trapped onto the filter membrane were stained in 1:20 Giemsa solution (Sigma, Taufkirchen, Germany). Counting was performed with a light microscope under ×10 magnification. After counting, depending on the availability of blood, a second filter was made for each sample with the aim to filter a volume of blood capturing 50, 100, 500, or 1,000 MF. This filter was kept at room temperature for later DNA extraction.

### Confirmation of *M. perstans* by PCR.

DNA was extracted from filters containing *M. perstans* MF using the QIAamp^®^ DNA Mini Kit (Qiagen, Hilden, Germany) according to the tissue protocol with the following changes: filters were incubated in lysis buffer overnight at 56°C and DNA was eluted in 50 µL of elution buffer.^26^ The DNA was kept at −20°C for long-term storage.

A duplex quantitative polymerase chain reaction (qPCR) was used for the confirmation of *M. perstans* and to rule out PCR inhibitors in the DNA.^28^ Two sets of primers were used: one targeted the internal transcribed spacer 1 (ITS1; GenBank: KJ631373) specific for *M. perstans* which consisted of Mp_ITS1_FW GGTGATATTCGTTGGTGTCTAT and Mp_ITS1_RV AGCTATCGCTTTATCTTCATCA. The second primer set targeted the murine IFN-γ gene (mIFN-γ; GenBank: AC_000032.1) to control for PCR inhibitors and consisted of mIFN-γ-FW TCAAGTGGCATAGATCTGGAAGAA and mIFN-γ-RV TGGCTCTGCAGGATTTTCATG. Hybridization probes were included for species-specific detection: Mp_ITS1 probe Fam-TCCAAATTATCGCCTAAACCGTCGA-TAMRA and mIFN-γ Hex-TCACCATCCTTTTGCCAGTTCCTCCAG-BHQ1. Primers and hybridization probes were purchased from biomers.net GmbH (Ulm, Germany).^28^

The PCR mix was 20 μL, composed of 1X QuantiNova buffer (Qiagen), 500 nM of each MpITS1 primer, 400 nM of each mIFN-γ primer, 50 nM of Mp_ITS1 hybridization probe, and 100 nM of mIFN-γ hybridization probe; plasmid DNA containing mIFN-γ (1 × 10^4^ copies/µL); and 2 μL of DNA as the template. The PCR run consisted of an initial heating phase of 95°C for 2 minutes followed by 35 cycles of denaturation (95°C for 10 seconds) and extension (62°C for 10 seconds) in a Rotor Gene 6000 cycler (Qiagen). Fluorescence was acquired at the end of each amplification step on the FAM and HEX channels. The samples were run in triplicate. A plasmid containing the ITS1 sequence was included in all runs as positive control for the real-time PCR, whereas water was included instead of DNA template to act as negative control to detect possible contamination. Samples in which the spiked mIFN-γ plasmid signal had *C*_t_ > 3 cycles beyond the no-template control were considered to contain inhibitors to the PCR. In such cases, DNA samples would be diluted until the inhibition was not seen. No samples showed any evidence of PCR inhibitors by this method.

To validate for specificity of the *M. perstans* PCR, it was tested against (unspecific) detection of *W. bancrofti*, a filarial nematode where MF would also be found in the blood of affected individuals. For this, *W. bancrofti* was extracted from 10 filters used for filtering blood of *W. bancrofti*–infected patients from earlier studies^27^ containing 80–1,000 MF/filter, according to the protocol for DNA purification from tissues from QIAamp DNA Mini Kit (Qiagen). PCR conditions for the *M. perstans* PCR were the same as mentioned earlier. None of the *W. bancrofti* samples gave a signal using the *M. perstans* ITS1 primers and hybridization probe.

### Quantification of *M. perstans* harboring *Wolbachia* by qPCR.

*Wolbachia* depletion was monitored by real-time PCR and a standard curve for the single copy number *ftsZ* gene. Two microliters of DNA, extracted from *M. perstans* MF on filters as described earlier, was used as template in a 20 µL reaction containing 1X QuantiNova buffer (Qiagen), 500 nM ftsZ_FW CGATGAGATTATGGAACA and ftsZ_RV TTGCAATTACTGGTGCTGC primers, and 25 nM hybridization probe ftsZ_FAM‐CAGGGATGGGTGGTGGTACTGGAA‐TAMRA. The PCR run consisted of an initial activation phase of 95°C for 2 minutes followed by 45 cycles of denaturation (95°C for 5 seconds) and extension (58°C for 30 seconds) in a Rotor Gene 6000 cycler (Qiagen), with fluorescence acquisition on the FAM channel at the end of each extension step. A plasmid containing 3.63 × 10^5^ *ftsZ* copies/µL was used as a positive control and to import the *ftsZ* standard curve for quantification. *FtsZ* copies were normalized to the number of MF that were deposited on the filters. Extrapolated *ftsZ*/MF less than the detection limit of 1.68 × 10^−1^ *ftsZ*/MF were set to 0.01 for statistical analyses.

### Statistical analyses.

The primary end point was based on MF levels at 12 months after study onset (12 months after treatment start for the early and 6 months after treatment start for the delayed treatment group). As secondary end points, MF loads were assessed at 4 months (4 months after treatment start for the early, 2 months before treatment start for the delayed treatment group) and at 24 months (24 months after treatment start for the early and 18 months after treatment start for the delayed treatment group) to determine the efficacy of doxycycline.

Two types of analysis were performed. Participants who were randomized and available for at least one follow-up were analyzed by intention to treat (ITT). Those who completed doxycycline daily for 6 weeks in their assigned treatment group and were available for the 4- and 12-month follow-up visits were analyzed by the per-protocol (PP) analysis. These two analyses were performed to ensure that the randomized study approach was not compromised.

For the statistical analyses, IBM SPSS software version 24 (IBM Corp., Ehningen, Germany) was used. Continuous variables are described with either mean ± standard deviation or median with minimum–maximum, and 25th and 75th percentiles; 95% CIs are shown for the mean (age, weight) or the median (MF, *Wolbachia ftsz*/MF). The 95% CIs for the median were calculated with bootstrapping. For MF counts, the geometric mean (GM) is shown to facilitate the comparability in meta-analyses. The GM was calculated by adding one to all values. After the calculation, one was subtracted from the result. Nominal variables (gender and MF positivity) are shown as numbers, in percent per total number and with 95% CIs.

The baseline characteristics of participants such as age and weight were compared using the *t*-test for independent variables between the early and the delayed doxycycline group. Gender distribution was compared using Fisher’s exact test.

Comparisons of MF loads and *W. ftsz*/MF between the early and delayed treatment groups were analyzed using the Mann–Whitney *U* test. Because data did not meet the assumptions required for parametric tests, nonparametric tests were performed. The Wilcoxon signed rank test was used to compare the differences in median between MF loads in the same treatment group at baseline versus the MF loads at 4, 12, and 24 months, respectively. Fisher’s exact test was used to compare the proportion of MF-positive and MF-negative individuals in both treatment groups at baseline and follow-ups. Comparisons between the proportions at baseline and the respective follow-ups in the same treatment group were carried out with the McNemar test. Two-tailed *P*-values < 0.05 were considered statistically significant. GraphPad Prism version 5.0 for Windows (www.graphpad.com, La Jolla, California) software was used for plotting the graphs.

## RESULTS

### Baseline characteristics.

Eligible participants were enrolled between November 2014 and June 2016. From 1,215 participants who were screened at the communities, 407 MF-positive patients were identified, but 189 were excluded at the community level based on age (*N* = 21), weight (*N* = 107), pregnancy (*N* = 5), breastfeeding (*N* = 36), or relocating (*N* = 20) criteria. Of 218 thought to be eligible, 16 were excluded for relocating (*N* = 5), alcohol dependence (*N* = 3), or abnormal liver function tests (*N* = 8). The prevalence of *M. perstans* MF, based on the screening, was 33%.

**Figure 1.**
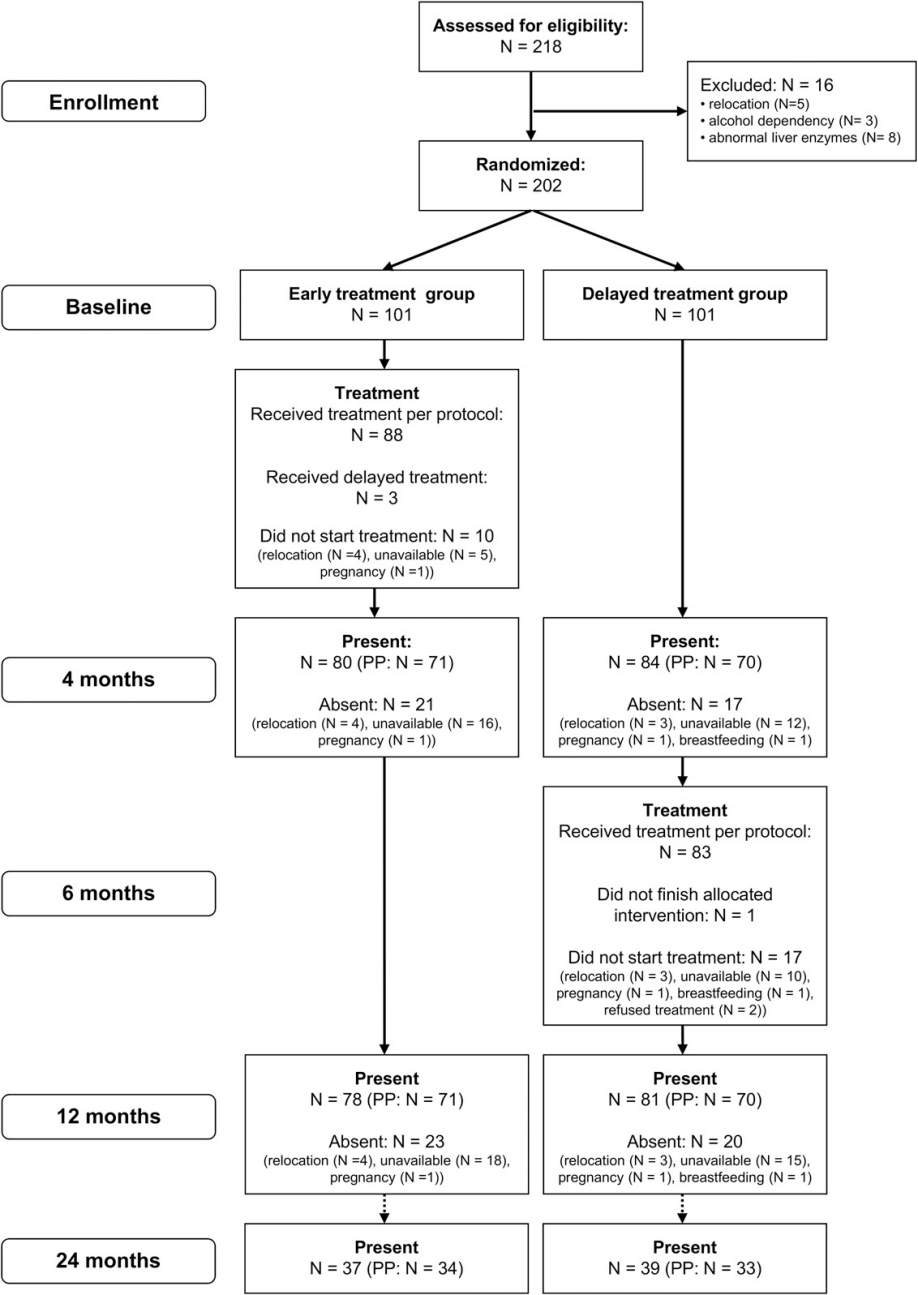
Recruitment and treatment profile of study participants.

The flow chart (Figure 1) shows that of 202 participants, 101 patients each were randomized into the two treatment arms. Of those participants randomized, 91 participants in the early and 84 in the delayed group received treatment. Of 171 patients who did take the treatment PP (early *N* = 88, delayed: *N* = 83), 141 patients were present at the 4- and 12-month follow-ups and had MF filter counts for all three time points (early: *N* = 71, delayed: *N* = 70). These 141 patients were assessed for the primary end point.

The mean age for the early treatment group was 32 ± 14 years and that for the delayed group was 33 ± 13 years (Table 1, *P* = 0.594). The mean body weight of the early (58 ± 11 kg) and the delayed group (56 ± 8 kg) was also not significantly different (*P* = 0.325). Gender distribution was similar between the two treatment groups. On average, participants had stayed in the study community for more than 13 years.

**Table 1:** Baseline data of all randomized participants (intention to treat)

			Early treatment	Delayed treatment	*P*-value
		*N*	101	101	
Gender	Female	*N* (% [95% CI])	36 (35.6% [26.8; 45.3])	37 (36.6% [27.7; 46.3])	*P* = 1.0*
	Male	*N* (% [95% CI])	65 (64.4% [54.7; 73.2])	64 (63.4% [53.7; 72.3])	
Age (years)		Mean ± SD [95% CI]	32 ± 14 [29; 34]	33 ± 13 [30; 35]	*P* = 0.594†
		Min–max	10–55	10–55	
		Median	31	33	
		Percentiles (25th; 75th)	19; 42	21; 42	
Weight (kg)		Mean ± SD [95% CI]	58 ± 11 [56; 60]	56 ± 8 [55; 58]	*P* = 0.325†
		Min–max	40–88	40–74	
		Median	57	57	
		Percentiles (25th; 75th)	50; 65	50; 62	
MF/mL (filter)‡		Median [95% CI]	108 [85; 161]	97 [51; 174]	*P* = 0.328§
		Geometric mean	130	98	
		Min–max	0–24,980	0–24,870	
		Percentiles (25th; 75th)	43; 558	20; 400	

MF = microfilariae.

* Fisher’s exact test.

† *t*-test for independent samples.

‡ Filter results are missing from one patient in the early group (*N* = 100).

§ Mann–Whitney *U* test.

### Adherence to treatment and dropout rate.

Ninety-one of the 101 participants who were randomized into the early treatment group received immediate treatment, whereas 84 of the 101 participants randomized to the delayed treatment group received the treatment 6 months after the early group. Participants who did not receive the study medication had relocated to new communities, traveled at the time of treatment, became pregnant, were breastfeeding, or refused the treatment (delayed group) (Figure 1). Three patients randomized to the early group received their treatment together with the delayed group. These three patients were excluded from the PP but were included in the ITT analysis. Adherence to treatment was very high with only one patient opting out during the delayed treatment. At the 12-month follow-up, a dropout rate of 23% was recorded in the early and 21% in the delayed treatment group (Figure 1).

### Adverse events experienced by participants during treatment.

All AEs occurred within the first week of treatment and included general body pains, dizziness, diarrhea, headache, nausea, abdominal pains, and vomiting. A total of 16 (9.2%) of 174 participants who started the treatment reported one or more AEs (Table 2). One participant reported having two AEs (nausea and abdominal pains, lasting for 2 days) on the third day of treatment. Other participants only reported one of the AEs. Twelve of the AEs occurred in the early treatment group and five in the delayed treatment group. The AEs with the longest reported duration, diarrhea and dizziness reported by two participants, lasted for 3 days. There was no serious AE reported during the study. All reported AEs resolved spontaneously without intervention, and none of the participants who reported an AE stopped taking the trial medication.

**Table 2:** Adverse events reported during treatment

Adverse events	No. of patients	Grade (*N*)	Relation to treatment (*N*)
Nausea	3	Grade 1 (3)	Possible (3)
Dizziness	3	Grade 1 (3)	Possible (1)
			Remote (2)
General body pain	3	Grade 1 (3)	Possible (1)
			Not related (2)
Abdominal pain	2	Grade 1 (2)	Possible (1)
			Remote (1)
Headache	2	Grade 1 (2)	Possible (2)
Bloody diarrhea	1	Grade 1 (1)	Possible (1)
Vomiting	1	Grade 1 (1)	Possible (1)
Diarrhea	1	Grade 1 (1)	Possible (1)
Cold	1	Grade 1 (1)	Remote (1)

### Comparison of MF loads.

**Figure 2.**
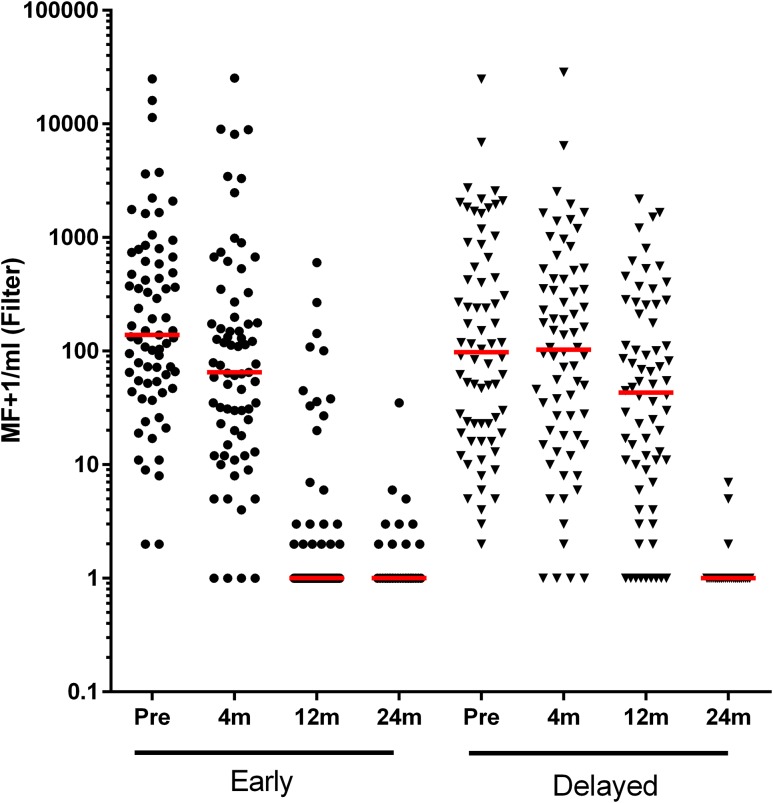
Changes in MF loads after doxycycline treatment—primary end point. Microfilariae were filtered onto 3 µM Whatman Nucleopore filters, Giemsa-stained, and counted as MF/mL of blood. Data were transformed by adding one to all values before plotting logarithmically. Comparison of MF/mL was performed within the treatment group at baseline and the 4-, 12-, and 24-month follow-up visits. The red horizontal line represents the median values in each group at each time point. Early and delayed refer to the staggered treatment of the two randomized groups. Early = the group that received treatment immediately after randomization. Delayed = the group that received treatment 6 months after randomization and acted as control to the Early group at the 4- and 12-month follow-up points. The graph was generated with GraphPad Prism 5.0 for Windows (La Jolla, CA). MF = microfilariae.

MF loads at baseline were similar between the early and delayed groups. At the 4-month follow-up, there was a statistically significant decrease in the MF load in the early group (*P* < 0.001; Table 3 [PP], Figure 2, Supplemental Table 1 [ITT]). The rather slow decrease of MF following doxycycline (e.g., as opposed to a microfilaricidal drug such as ivermectin) is reminiscent of the slow MF decline 4–6 months after doxycycline treatment in *W. bancrofti* or *Onchocerca volvulus* infections.^27,29^

**Table 3:** Microfilariae counts (Nucleopore filter, Giemsa-stained)—per protocol analysis

		Early treatment	Delayed treatment	*P*-value
MF-positive baseline	MF+/total (% [95% CI])	71/71 (100%)	70/70 (100%)	cannot calculate
MF-positive 4 months	MF+/total (% [95% CI])	67/71 (94.4% [87.2; 98.1])	66/70 (94.3% [87.0; 98.0])	*P* = 1.0*
MF-positive 12 months	MF+/total (% [95% CI])	23/71 (32.4% [22.4; 43.8])	61/70 (87.1% [77.9; 93.4])	*P* < 0.001*
MF-positive 24 months	MF+/total (% [95% CI])	10/34 (29.4% [16.2; 45.9])	3/33 (9.1% [2.6; 22.3])	*P* = 0.062*
MF/mL baseline	*N*	71	70	*P* = 0.179†
	Median [95% CI]	138 [94; 329]	97 [50; 239]	
	Geometric mean	175	112	
	Min–max	1–24,980	1–24,869	
	Percentiles (25th; 75th)	54; 602	22; 548	
MF/mL 4 months	*N*	71	70	*P* = 0.454†
	Median [95% CI]	64 [34; 124]	102 [49; 189]	
	Geometric mean	76	89	
	Min–max	0–25,300	0–28,480	
	Percentiles (25th; 75th)	18; 188	17; 428	
Comparison with baseline	*P*-value‡	*P* < 0.001	*P* = 0.05	
MF/mL 12 months	*N*	71	70	*P* < 0.001†
	Median [95% CI]	0	42 [16; 76]	
	Geometric mean	1.3	34	
	Min–max	0–601	0–2,176	
	Percentiles (25th; 75th)	0; 1	8; 211	
Comparison with baseline	*P*-value‡	*P* < 0.001	*P* < 0.001	
MF/mL 24 months	*N*	34	33	*P* = 0.045†
	Median [95% CI]	0	0	
	Geometric mean	0.5	0.1	
	Min–max	0–34	0–6	
	Percentiles (25th; 75th)	0; 1	0; 0	
Comparison with baseline	*P*-value‡	*P* < 0.001	*P* < 0.001	

MF = microfilariae.

* Fisher’s exact test.

† Mann–Whitney *U* test.

‡ Wilcoxon signed rank test.

Looking at the PP analysis at the 12-month follow-up, only 32% of the participants still had (few) MF and the overall median MF load had reduced significantly to 0 MF/mL (*P* < 0.001) in the early group. Of the 23 (32%) persons with MF at this time point, 12 had ≤ 10 MF/mL. Within the delayed group, in which doxycycline treatment was deferred for 6 months, 87% still had MF at 12 months, significantly higher than that in the early group (*P* < 0.001). Of the 61 (87%) persons with MF at this time point, only 13 had ≤ 10 MF/mL. The median MF (42 MF/mL) was still significantly higher than that in the early group (*P* < 0.001). An equivalent, significant difference between early and delayed groups, regarding both proportions of MF-positive individuals and median microfilaremias, was observed when the ITT groups were analyzed (Table 3 [PP], Supplemental Table 1 [ITT]).

In conclusion, these data show a clear and significant influence of doxycycline treatment after 4 months and more so after 12 months on MF loads in the early group, whereas the decline in MF loads in the delayed group lagged behind, as expected. This is similar to what is known from *W. bancrofti* and *O. volvulus* infections treated with doxycycline^27,30–33^ and, together with the data on *Wolbachia* loads (see following paragraphs), strongly argues for an anti-wolbachial effect of doxycycline.

### Subgroup analysis of patients present through month 24.

Because of civil unrest in some of the communities, only approximately 40% percent of the patients could be followed up through 24 months. A total of 76 participants, 37 in the early group and 39 in the delayed group, donated blood for the MF analysis. In both early and delayed groups, the median microfilaremia remained 0 MF/mL (Table 3 [PP]; Supplemental Table 1 [ITT]).

In conclusion, the effect of doxycycline manifests within a few months after treatment as a long-term absence of microfilaremia in the treated patients.

### Secondary outcomes.

#### Mansonella perstans ITS1 *PCR analysis*.

To control the microscopic analysis of MF, real-time PCR that amplifies an *M. perstans* gene was performed using blood samples that had been microscopically examined to be positive (*N* = 107) or negative (*N* = 2) for *M. perstans*. The 107 MF-positive samples were confirmed to be *M. perstans* by amplification of the *M. perstans* ITS1 amplicon and the two *M. perstans*–negative MF samples were confirmed to be *M. perstans* negative by no amplification of the ITS1 amplicon. The samples contained MF ranging from 1 to 11,430 MF/mL. *Wuchereria bancrofti–*positive MF (500 MF/filter) obtained from 10 control filters were PCR negative (Supplemental Table 2).

### Quantification of *M. perstans Wolbachia* in MF after doxycycline treatment.

After quantifying MF, a second filter was made at baseline and the 4- and 12-month follow-up time points from participants for which enough blood was available. DNA was extracted from the MF on the second filter and the *Wolbachia* content quantified by qPCR using the single copy number *ftsZ* gene normalized to MF (*ftsZ*/MF). In the PP analysis, at the 4-month follow-up, the early group had significantly fewer samples positive for *Wolbachia* than the delayed group (18/49 versus 38/48, *P* < 0.001, Table 4) and the early group baseline values (18/49 versus 50/63, *P* < 0.001). At month 12, significantly fewer samples from the delayed group, which had had treatment 6 months before this follow-up, were positive for *Wolbachia* than the baseline values (18/39 versus 47/63, *P* = 0.004). No significant difference between the early and delayed groups at the 12-month follow-up was seen (7/9 versus 18/39, *P* = 0.14).

**Table 4:** Detectable *Wolbachia* in PCR—signal above detection limit

		Early treatment	Delayed treatment	*P*-value
*Wolbachia* detectable baseline	PCR+/total (% [95% CI])	50/63 (79.4% [68.2; 87.9])	47/63 (74.6% [62.9; 84.1])	*P* = 0.673*
*Wolbachia* detectable 4 months	PCR+/total (% [95% CI])	18/49 (36.7% [24.3; 50.7])	38/48 (79.2% [66.2; 88.8])	*P* < 0.001*
Comparison with baseline	*P*-value†	*P* < 0.001	*P* = 1.0	
*Wolbachia* detectable 12 months	PCR+/total (% [95% CI])	7/9 (77.8% [45.6; 95.1])	18/39 (46.2% [31.3; 61.6])	*P* = 0.14*
Comparison with baseline	*P*-value†	cannot calculate	*P* = 0.004	

* Fisher’s exact test.

† McNemar’s test.

In the early group, median *ftsZ* copies/MF significantly decreased 24-fold from a median of 0.24 at baseline to 0.01 by month 4 after the beginning of the treatment (Table 5, *P* < 0.001; ≥ 1-log drop). At the 12-month follow-up, *Wolbachia* depletion was still ≥ 1 log lower than that at baseline, 0.01 versus 0.24, respectively (Table 5, *P* < 0.001). At the 4-month follow-up, the delayed group median *ftsZ* copies/MF decreased slightly compared with the baseline from a median of 0.27 to 0.17 (Table 5, *P* = 0.031), even though this group had not yet received the treatment. Although formally significant, this is well within the range of baseline values. At the 12-month follow-up (6 months after treatment start), the delayed group median *ftsZ* copies/MF had decreased from 0.27 to 0.1 (Table 5, *P* < 0.001; ≥ 1-log drop). Comparing the early treatment group with the delayed treatment group, no significant difference between the baseline *ftsZ* copies/MF was seen (*P* = 0.734), whereas at the 4-month follow-up, the early group that had received treatment had significantly lower *ftsZ* copies/MF than the delayed group (Table 5; 0.01 versus 0.17, *P* < 0.001). At the 12-month follow-up, at least 6 months after both groups had received treatment, the expected biologically relevant decline in *ftsZ* copies/MF of 0.01 was achieved in the delayed group (Table 5).

**Table 5:** *Wolbachia ftsZ*/MF–per-protocol analysis*

		Early treatment	Delayed treatment	*P*-value
*Wolbachia*/MF baseline	*N*	63	63	*P* = 0.734†
	Median [95% CI]	0.24 [0.17; 0.62]	0.27 [0.17; 0.65]	
	Min–max	0.01–13.1	0.01–38.8	
	Percentiles (25th; 75th)	0.17; 1.91	0.09; 1.62	
*Wolbachia*/MF 4 months	*N*	50	50	*P* < 0.001†
	Median [95% CI]	0.01 [0.01; 0.09]	0.17 [0.17; 0.17]	
	Min–max	0.01–0.52	0.01–12.8	
	Percentiles (25th; 75th)	0.01; 0.17	0.17; 0.3	
Comparison with baseline	*P*-value‡	*P* < 0.001	*P* = 0.031	
*Wolbachia*/MF 12 months	*N*	48	44	*P* = 0.015†
	Median [95% CI]	0.01 [0.01; 0.01]	0.01 [0.01; 0.17]	
	Min–max	0.01–1.77	0.01–2.53	
	Percentiles (25th; 75th)	0.01; 0.01	0.01; 0.17	
Comparison with baseline	*P*-value‡	*P* < 0.001	*P* < 0.001	

MF = microfilariae.

* Values for MF-negative patients and values below the real-time PCR detection limit were set to 0.01.

† Mann–Whitney *U* test.

‡ Wilcoxon signed rank test.

Essentially equivalent results to the PP analysis were obtained when analyzing the ITT data from both groups (Supplemental Tables 3a and 3b). From PP and ITT analyses, *Wolbachia* were depleted by ≥ 1 log 4 months (early group) and 12 months (early and delayed groups) after the study start compared with baseline values, indicating anti-wolbachial efficacy of doxycycline 200 mg/day for 6 weeks.

## DISCUSSION

In contrast to other filarial diseases such as onchocerciasis caused by *O. volvulus* treated with ivermectin and lymphatic filariasis caused by *W. bancrofti* treated with ivermectin and/or DEC plus albendazole, *M. perstans* infection is not treated effectively with these drugs.^1,5^ However, one aspect of the biology of filarial nematodes that could be exploited for the treatment and control of mansonellosis is the presence of the endosymbiotic *Wolbachia* found in several filarial species, including *W. bancrofti*, *O. volvulus*,^34^ and *M. perstans*,^22–24^ but not in *Loa loa.*^34^ Studies of symbiotic *Wolbachia* organisms suggest that these endobacteria are important as both chemotherapeutic targets and disease-causing organisms.^35^ Therefore, the discovery that *M. perstans* from Mali^22,23^ were positive for *Wolbachia*, and thus treatable by doxycycline,^23^ was a major advance for treating mansonellosis.^6^

In the present randomized, open-label trial, we have shown that a 6-week course of doxycycline is effective in mediating long-term reductions of microfilaremia in a Ghanaian population infected with *M. perstans*. Here, we describe an almost complete loss of microfilaremia 12 months after doxycycline treatment, which was maintained up to 24 months. Following doxycycline treatment, we recorded a ≥ 1-log drop in *Wolbachia* load in the early and delayed groups 4 and 6 months after treatment, respectively. At the 12-month follow-up, there was no significant difference in the number of samples positive for *Wolbachia* between the early and delayed groups. This is probably an effect of the low *N* of participants who were MF positive at this time in the early group and the anti-wolbachial effect of doxycycline treatment that occurred 6 months before this follow-up in the delayed group. Compared with baseline within the groups, a significant reduction in the number of samples positive for *Wolbachia* PCR can be seen at the 4-month follow-up in the early group and at the 12-month follow-up in the delayed group (i.e., 6 months after treatment). The absence of MF in doxycycline-treated patients^33^ is most likely due to the effect of *Wolbachia* depletion on embryogenesis and decline of MF from host blood through natural attrition, as reported in onchocerciasis^27,30,36^ and lymphatic filariasis,^26,27,32^ and supports the results from a study on *M. perstans* in Mali.^23^

Within the group followed for 24 months as secondary end point, one participant from the early group had 2,091 MF/mL at baseline and zero at the 12-month follow-up but had 5 MF/mL at the 24-month follow-up. This might have been due to new infection or the limitations of MF assessment/applied methods, especially in individuals with low MF counts.

Reinfection is likely to occur in this area of ongoing transmission, given that it is estimated that the adult worms are long-lived (MF can be detected 10 years after leaving an endemic area).^37^ New infections with concurrent rise in *Wolbachia* levels following doxycycline treatment have been documented for onchocerciasis, where old, doxycycline-treated, and thus *Wolbachia*-depleted, female worms were located in onchocercomata next to young, nulliparous worms that were full of *Wolbachia*.^31^ Because of the unavailability of adult worms for histological analysis in *M. perstans* infection, we could not prove these findings for mansonellosis in a similar manner. There is the need for continued study to determine how long it takes for reinfection to occur in *M. perstans* infections. However, a slight increase in MF levels (not significant) at month 24 in the early group, that is, the group with the longest time difference between treatment and follow-up, may support the occurrence of new infection in our study group(s).

Doxycycline was well tolerated as in all other previous treatments.^26,27,30,32,36^ Sixteen participants reported having AEs. One patient reported two AEs. Recorded AEs–dizziness, diarrhea, headache, nausea, abdominal pains, and vomiting—were not above grade three and were AEs associated with doxycycline when patients do not eat before taking drugs. All the AEs occurred in the first week of the treatment and resolved without treatment interruption.

There have been conflicting reports with regard to the presence of *Wolbachia* endosymbionts in the MF of *M. perstans* from Gabon^24,25^ and Mali.^22,23^ This presents a controversial argument for the use of doxycycline as treatment of *M. perstans* infections.^38^ It is unclear whether these differences are due to methodological limitations of the methods used or whether this indicates that different strains of *M. perstans* exist in different parts of Africa. Our study supports the study by Coulibaly et al.^23^ in which doxycycline is effective in treating mansonellosis. Both studies demonstrated that doxycycline is effective in depleting *Wolbachia*, at least from MF, and that subsequent to the depletion of the endobacteria, MF levels are reduced. This reduction is most likely due to the block of embryogenesis, as shown for *W. bancrofti* by PCR and for *B. malayi* and *O. volvulus* by histology.^39^ As with *W. bancrofti*, we could not conclusively show this because we could not retrieve adult worms from treated individuals. The direct link between *Wolbachia* and *M. perstans* can only be shown when there is an animal model for the life cycle. Such a model will also clarify the distribution of *Wolbachia*/MF, that is, whether all MF are carriers of the endobacteria and *Wolbachia* numbers are equivalent in different MF.

Currently, doxycycline is not recommended for mass treatment because of logistical difficulties, such as the cost of delivery of the drug and contraindication in pregnant women, lactating mothers, and children younger than 10 years. However, this treatment should be explored for *M. perstans* control in special situations. It could be used as a curative treatment for individuals wanting to remain free of infection for a long term.^40^ As the doxycycline regimen of 6 weeks that results in nearly complete MF clearance is too long for mass treatment, there remains the need to explore further drug combinations with other already registered drugs and new anti-wolbachial antibiotics. The value of this trial is in the proof of concept and showing a possibility for those individual patients who seek treatment because of symptoms.

## CONCLUSION

This study shows that *M. perstans* filarial nematodes in Ghana harbor *Wolbachia* endobacteria. These endosymbionts can be depleted by treatment with 200 mg/day doxycycline for 6 weeks. Subsequent to doxycycline treatment, there is a gradual and permanent reduction of MF levels as has been shown for other filarial nematodes that have been shown to be dependent on *Wolbachia* endobacteria.

## Supplementary Files

Supplemental tables
