# Supplementary material for: The Efficacy of Doxycycline Treatment on Mansonella perstans Infection: An Open-Label, Randomized Trial in Ghana
Source: Am J Trop Med Hyg. 2019 Jun 3;101(1):84–92. doi: 10.4269/ajtmh.18-0491 (PMC6609185; doi:10.4269/ajtmh.18-0491)
Supplement: Supplementary file 1 [file tpmd180491.SD1.pdf]

Supplementary Tables

Supplementary table 1. MF-counts (Nucleopore filter, Giemsa stained) – ITT analysis

		**Early treatment**	**Delayed treatment**	*p-value*
**MF positive baseline**	MF +/total (% [95% CI])	99/100 (99% [95.4; 99.9])	100/101 (99% [95.5; 99.9])	*p = 1.0*[c]
**MF positive 4months**	MF +/total (% [95% CI])	76/80 (95% [88.6; 98.3])	79/84 (94% [87.4; 97.7])	*p = 1.0*[c]
Comparison to baseline	*p-value*[a]	*p = 0.375*	*p = 0.125*	
**MF positive 12 months**	MF +/total (% [95% CI])	25/77 (32.5% [22.8; 43.4])	70/80 (87.5% [79.0; 93.4])	*p < 0.001*[c]
Comparison to baseline	*p-value*[a]	*nd*	*p = 0.004*	
**MF positive 24 months**	MF +/total (% [95% CI])	11/37 (29.7% [16.9; 45.6])	6/39 (15.4% [6.7; 29])	*p = 0.172*
Comparison to baseline	*p-value*[a]	*nd*	*nd*	
**MF/ml baseline**	Median [95% CI]	108 [85; 161]	97 [51; 174]	
	Geometric mean	130	98	
	Min - Max	0 - 24980	0 - 24870	
	Percentiles (25th;75th)	43; 558	20; 400	*p = 0.312*[d]
**MF/ml 4 months**	Median [95% CI]	61 [30; 117]	102 [53; 183]	
	Geometric mean	65	85	
	Min - Max	0 - 25300	0 - 28480	
	Percentiles (25th;75th)	13; 173	17; 380	*p = 0.256*[d]
Comparison to baseline	*p-value*[b]	*p <0.001*	*p = 0.067*	
**MF/ml 12 months**	Median [95% CI]	0	40 [16; 72]	
	Geometric mean	1	33	
	Min - Max	0 - 601	0 - 2176	
	Percentiles (25th;75th)	0; 1	7; 233	*p < 0.001*[d]
Comparison to baseline	*p-value*[b]	*p <0.001*	*p <0.001*	
**MF/ml 24 months**	Median [95% CI]	0	0	
	Geometric mean	0.5	0.4	
	Min - Max	0 - 34	0 - 101	
	Percentiles (25th;75th)	0; 1	0; 0	*p = 0.19*[d]
Comparison to baseline	*p-value*[b]	*p <0.001*	*p <0.001*	

[a] McNemar-test [b] Wilcoxon signed rank test [c] Fisher's exact test

[d] Mann-Whitney-U test

Supplementary table 2. Confirmation of *M. perstans* in blood of study participants using MpITS1 real-time PCR

	**PCR positive/total (%)**
***M. perstans* infected blood sediment**	107/109 (98.2%)
***W. bancrofti* filters**	0 /10 (0%)

Abbreviations: MF, Microfilarae; pos, positive; neg, negative.

Supplementary table 3a. **Detectable *Wolbachia* in PCR – ITT analysis**

		Early treatment	Delayed treatment	*p-value*[d]
***Wolbachia* detectable baseline**	PCR+/total (% [95% CI])	69/91 (75.8% [66.3; 83.7])	71/90 (78.9% [69.6; 86.3])	*p = 0.723*[c]
***Wolbachia* detectable 4 months**	PCR+/total (% [95% CI])	21/57 (36.8% [25.2; 49.8])	50/62 (80.6% [69.5; 89])	*p < 0.001*[c]
Comparison to baseline	*p-value*[a]	*p < 0.001*	*p = 0.824*	
***Wolbachia* detectable 12 months**	PCR+/total (% [95% CI])	9/12 (75% [47.1; 92.4])	24/46 (52.2% [38; 66.1])	*p = 0.201*[c]
Comparison to baseline	*p-value*[a]	*p = 1.0*	*p = 0.007*	

[a] McNemar-test [c] Fisher's exact test

Supplementary table 3b. ***Wolbachia ftsZ*/MF – ITT analysis***

		Early treatment	Delayed treatment	*p-value*[d]
***Wolbachia ftsZ*/MF baseline**	N	91	91	
	Median [95% CI]	0.24 [0.17; 0.57]	0.3 [0.17; 0.57]	
	Min - Max	0.01 - 13.1	0.01 - 38.8	
	Percentiles (25th;75th)	0.17; 1.91	0.17; 1.62	*p = 0.952.*
***Wolbachia ftsZ*/MF 4 months**	N	61	67	
	Median [95% CI]	0.01 [0.01; 0.01]	0.17 [0.17; 0.17]	
	Min - Max	0.01 - 0.52	0.01 - 12.8	
	Percentiles (25th;75th)	0.01; 0.17	0.09; 0.17	*p < 0.001*
Comparison to baseline	*p-value*[b]	*p < 0.001*	*p = 0.021*	
**Wolbachia *ftsZ*/MF 12 months**	N	62	56	
	Median [95% CI]	0.01 [0.01; 0.01]	0.01 [0.01; 0.17]	
	Min - Max	0.01 - 1.77	0.01 - 2.53	
	Percentiles (25th;75th)	0.01; 0.01	0.01; 0.17	*p = 0.002*
Comparison to baseline	*p-value*[b]	*p < 0.001*	*p < 0.001*	

*Values for Mf-negative patients and values under detection limit were set to 0.01

[b] Wilcoxon signed rank test [d] Mann-Whitney-U test
